# Supplementary material for: Unique Severe COVID-19 Placental Signature Independent of Severity of Clinical Maternal Symptoms
Source: Viruses. 2021 Aug 23;13(8):1670. doi: 10.3390/v13081670 (PMC8402730; doi:10.3390/v13081670)
Supplement: Supplementary file 1 [file viruses-13-01670-s001.zip › viruses-1323196 suppl.pdf]

Supplementary data S1

Overview of clinical presentation of COVID-19 in cases of confirmed placental SARS-CoV-2

Case 1

The first of the four immunohistochemical SARS-CoV-2 positive placentas was from a 40-year-old obese (BMI 36.4 kg/m^2)) woman with gestational diabetes who presented with premature rupture of membranes at 33+3 weeks of gestation. She tested positive for SARS-CoV-2 at 33+1 weeks of gestation. At clinical presentation, fetal cardiotocography (CTG) showed signs of fetal distress, including tachycardia and repetitive decelerations for which an emergency caesarean section was performed. A daughter was born with an Apgar score of 2, 5 and 8 at, respectively, 1, 5 and 10 minutes postpartum. The umbilical cord blood pH was 7.11 and the birthweight was 2445 grams (81th percentile). The SARS-CoV-2 RT-PCR of the vagina, urine, maternal and fetal side of the placenta were all positive.

Case 2

The second immunohistochemical SARS-CoV-2 positive placenta was from a woman with SARS-CoV-2 at 37+2 weeks of gestation who showed mild symptoms. She had an uncomplicated, spontaneous vaginal birth at 40+4 weeks gestation. A healthy neonate was born with an Apgar score of 10, 10 and 10 after respectively 1, 5 and 10 minutes. The umbilical cord blood pH was not obtained. The birthweight was 3185 grams (18th percentile). No swabs were obtained after birth.

Case 3

The third case was a woman who presented with decreased fetal movements at 31+5 weeks of gestation. The fetal CTG showed signs of fetal distress for which an emergency caesarean section was performed. A daughter was born with an Apgar score of 1, 3 and 7 at, respectively 1, 5 and 10 minutes postpartum. The umbilical cord blood pH was 7.14 and the birthweight was 1625 grams (26th percentile). Three days before presentation at the hospital she reported a fever for which no SARS-CoV-2 testing was performed. Due to these complaints and an unexplained abnormal CTG, SARS-CoV-2 PCR of the nasopharynx was performed just before the emergency caesarean section which turned out to be positive. No other PCR samples were collected during delivery due to the delayed diagnosis.

Case 4

The fourth case concerns a patient with mild clinical respiratory symptoms a week before presentation. This patient, an obese (BMI 30.1) women with gestational diabetes, presented with reduced fetal movements at 31+4 weeks of gestation. The CTG showed signs of fetal distress for which an emergency caesarean section was performed. A daughter was born with an Apgar score of 1, 4 and 6 at, respectively, 1, 5 and 10 minutes postpartum. The umbilical cord pH was 6.90 and the birthweight was 1880 grams (75th percentile). This case has previously been discussed in detail. (24) Of the PCR swabs sampled after birth, the PCRs of the vagina, urine, maternal and fetal side of the placenta all tested SARS-CoV-2 positive.

Except for the above-mentioned SARS-CoV-2 positive PCR swabs of the placenta (case 1 and case 4), no other positive placental PCR swabs were found in our study population.
